# Exposure to a Titanium Dioxide Product Alters DNA Methylation in Human Cells

**DOI:** 10.3390/nano14242037

**Published:** 2024-12-19

**Authors:** Carlos Wells, Marta Pogribna, Arjun Sharmah, Angel Paredes, Beverly Word, Anil K. Patri, Beverly Lyn-Cook, George Hammons

**Affiliations:** 1 Division of Biochemical Toxicity, FDA/National Center for Toxicological Research, Jefferson, AR 72079, USA; carlos.wells@fda.hhs.gov (C.W.); marta.pogribna@fda.hhs.gov (M.P.); beverly.word@fda.hhs.gov (B.W.); beverly.lyn-cook@fda.hhs.gov (B.L.-C.); 2 Division of Nanotechology Core, FDA/National Center for Toxicological Research, Jefferson, AR 72079, USA; arjun.sharmah@fda.hhs.gov (A.S.); angel.paredes@fda.hhs.gov (A.P.); anil.patri@fda.hhs.gov (A.K.P.)

**Keywords:** DNA methylation, titanium dioxide, human cells, nanotoxicity

## Abstract

The safety of titanium dioxide (TiO_2_), widely used in foods and personal care products, has been of ongoing concern. Significant toxicity of TiO_2_ has been reported, suggesting a risk to human health. To evaluate its potential epigenotoxicity, the effect of exposure to a TiO_2_ product to which humans could be exposed on DNA methylation, a primary epigenetic mechanism, was investigated using two human cell lines (Caco-2 (colorectal) and HepG2 (liver)) relevant to human exposure. Global methylation was determined by enzyme-linked immunosorbent assay-based immunochemical analysis. Gene promoter methylation was evaluated using EpiTect Methyl II Signature PCR System Array technology. Expression of DNA methyltransferases, *MBD2*, and *URHF1* was quantified by qRT-PCR. A decrease in global DNA methylation was observed in both cell lines. Across the cell lines, seven genes (*BNIP3*, *DNAJC15*, *GADD45G*, *GDF15*, *INSIG1*, *SCARA3*, and *TP53*) were identified in which promoters were methylated. Changes in promoter methylation were associated with gene expression. Results also revealed aberrant expression of regulatory genes, DNA methyltransferases, MBD2, and UHRF1. Findings from the study clearly demonstrate the impact of TiO_2_ exposure on DNA methylation in two cell types, supporting the potential involvement of this epigenetic mechanism in its biological responses. Hence, epigenetic studies are critical for complete assessment of potential risk from exposure.

## 1. Introduction

Titanium dioxide (TiO_2_) is widely used in the food industry, as well as in personal care products, including toothpastes, lip balms, shampoos, deodorants, and sunscreens, to provide a whitening/brightening effect [1,2]. The material is referred to as E171 in Europe and as INS171 in North America. In the USA, TiO_2_ can be used in food if its content does not exceed 1% of the total weight of the product [3]. TiO_2_ is added to many foodstuffs, including cheeses and sauces, skimmed milk, ice cream, and confectionery products, e.g., as coating on sweets and chewing gum [4,5,6,7,8,9]. Compared to other products, its content in sweets, and in particular candy, chewing gum, chocolate, and white-coated products, can be very high, reaching 2.5 mg titanium (Ti)/g of food.

Human exposure to TiO_2_ has been estimated in several populations. In the USA, the estimated average exposure of children below 10 years of age is 1–2 mg TiO_2_/kg body weight (bw)/day and above 10 years old is 0.2–0.7 mg TiO_2_/kg bw/day. In the UK, the estimated exposure is on average 2–3 mg TiO_2_/kg bw/day for children younger than 10 years and 1 mg TiO_2_/kg bw/day for children over 10 years of age [4]. A study performed in the Netherlands showed an intake of TiO_2_ in the same range as in the USA [10]. This study estimated the mean average exposure to be 0.67 mg TiO_2_/kg bw/day in children between 2 and 6 years old, 0.17 mg TiO_2_/kg bw/day in individuals between 7 and 69 years old, and 0.06 mg TiO_2_/kg bw/day in individuals above 70 years old. Also, long-term repeated exposure to such quantities of TiO_2_ may result in tissue accumulation [11]. The main physiological routes of TiO_2_ uptake are inhalation, ingestion, and dermal absorption. While inhalation may be the primary route for TiO_2_ exposure in the workplace, oral exposure, e.g., from food and personal care products, seems to be the most important source of TiO_2_ in non-occupational settings [10].

The safety of TiO_2_ has been repeatedly questioned in recent years by the scientific community and consumer associations [2,12]. TiO_2_ can consist of nanoparticles and microparticles as a consequence of its production process, which unintentionally generates particles of a broad size distribution [4,13,14,15]. Although the presence of nanoparticles in the material has raised particular concerns regarding its potential impact on human health, the importance of assessing TiO_2_ products as a whole has been noted, given the substantial difference from the reference material P25 and products to which humans are exposed in their diet and in the use of various personal products [16,17,18]. Several reports on TiO_2_ products showed no adverse effects; however, studies investigating the effects in vitro and in vivo in various animal and cell models have also discovered potential adverse effects, including the induction of inflammation, hepatotoxic effects, the formation of reactive oxygen species (ROS), and genotoxic effects, among others [19,20,21]. The potential harm of TiO_2_ products is not yet completely understood.

Most studies on the toxicity mechanisms of nanomaterials have focused on the effects of these materials on cells at the transcriptome and proteome levels. However, there is a critical need to also understand the epigenetic action of potential toxicants. Epigenetics is defined as “a stably heritable phenotype resulting from changes in a chromosome without alterations in the DNA sequence” [22]. DNA methylation is a major epigenetic mechanism that involves adding a methyl group to the fifth position of cytosine residue of CpG dinucleotides, resulting in 5′-methylated cytosine [23,24]. Methylation/demethylation of CpG sites is important for maintaining cell- or tissue-specific gene expression. Methylation of cytosines within CpG sequences and their subsequent interaction with methyl-CpG binding proteins (MBDs) may induce chromatin conformational modifications and inhibit the access of the transcriptional machinery to gene promoter regions, thus altering gene expression levels and playing a critical role in the regulation of numerous cellular processes [25]. Other binding proteins may be involved, such as UHRF1, which interacts with DNMT1 and is implicated in the maintenance of DNA methylation [26]. Promoter hypermethylation is commonly associated with gene silencing [27]. DNA methylation is established and maintained by DNA methyltransferases (DNMT1, DNMT3a, and DNMT3b) [28,29,30,31]. In particular, DNMT1 is the maintenance methylase that works explicitly on hemi-methylated DNA strands, whereas de novo methylation, the establishment of new methylation, is carried out by DNMT3a and DNMT3b.

The critical role of aberrations in DNA methylation in cancer has been well established [32,33,34]. Several types of aberration in the DNA methylation machinery occur, including global hypomethylation, hypermethylation of tumor suppressor genes, and aberrant expression of DNMTs. Additionally, aberrant DNA methylation is associated with complex non-malignant diseases, such as schizophrenia, major depression, hypertension, type-2 diabetes, and autoimmune and cardiovascular diseases [35,36,37]. Since alterations in DNA methylation have a critical role in the development and progression of numerous pathological states and diseases, as well as the recognition that epigenetic regulatory mechanisms can change dynamically in response to environmental stressors [38], including DNA methylation effects as part of the risk assessment of the toxicological potential of nanomaterials is rational. Studying changes in this epigenetic pathway should also allow better understanding of their toxicological mechanisms.

In previous studies, exposure to TiO_2_ nanoparticles, including the P25 model, has been shown to induce alterations in DNA methylation in target tissues and cells, even at low sub-cytotoxic doses, thus indicating that the epigenetic changes induced by TiO_2_ nanoparticles may be a critical contributor to the potential harm of these particles to human health [39,40,41,42]. These studies have, however, yet to include a commercially available TiO_2_ product to which humans may be exposed in their diet. The present study therefore examined the effects of such material. The assessment included changes in global methylation, gene-specific methylation, and expression levels of DMNTs, MBD2, and UHRF1. Array analysis of gene-specific methylation focused on genes indicative of cellular stress responses and toxicity. Expression levels of several genes whose promoter methylation status was altered were also determined.

A primary toxicity mechanism thought to be induced by TiO_2_ nanomaterials is oxidative stress [9,14,43,44,45]. Also, several studies have shown that a strong link exists between oxidative stress and the epigenetic process in various pathophysiological states [46], including cancer [47,48,49], diabetes [50], and exposure to various xenobiotics [51]. The results from the current study found that TiO_2_ exposure affected DNA methylation, both global and gene-specific, in the cell lines, supporting the involvement of DNA methylation in the potential toxicity of this material and the critical need for its epigenetic assessment.

## 2. Materials and Methods

### 2.1. Nanomaterial Source and Characterization

TiO_2_ was supplied by Pure Organic Ingredients (Linton, UT, USA) (product described as exceptionally pure) and purchased on Amazon.com. The nanomaterial was characterized using X-ray diffraction (XRD), Raman spectroscopy, scanning electron microscopy–energy-dispersive X-ray spectroscopy (SEM–EDS), transmission electron microscopy (TEM), and laser diffraction. The X-ray diffraction pattern of the powder sample of TiO_2_ was obtained using a Bruker D2 Phaser (Karlsruhe, Germany) operating at 30 kV and 10 mA to determine crystal form and structure. To prepare the sample for XRD, a finely powdered sample was loaded within the 25 mm-diameter well of a 51.5 mm-diameter PMMA ring. The sample was pressed flat using a glass slide such that the sample was approximately coplanar with the top of the PMMA ring. Any sample outside the well was cleaned with Kimwipes (Sacramento, CA, USA). Raman spectroscopy (Horiba T64000 LabRAM confocal Raman microscope (Rue de Lille, France) equipped with a 488 nm laser) was also used to determine the crystal form of the sample. The sample was prepared on a glass slide as a roughly flat, uniform, and thin layer of the powdered sample (about 50 mg) by pressing with another glass slide of approximately 10 mm × 10 mm size. Elemental composition was studied by scanning electron microscopy—energy-dispersive X-ray spectroscopy (SEM–EDS) (Zeiss Merlin scanning electron microscope (Jena, Germany) equipped with Ametek EDAX operating (Pleasanton, CA, USA) at 7 kV). Two different samples were prepared by mounting powdered samples on a Si tape and a C tape on a SEM stub. The samples were then sputter-coated with Au and Pd to improve conductivity and avoid charging the sample surface. EDS spectra and elemental mapping for samples on C tape and Si tape were obtained. Size distribution analysis was performed by TEM (Delong Instruments low-voltage electron microscope (LVEM25) (Brno, Czech Republic) at 25 kV equipped with sCMOS camera of sensor size 2560 × 2160 pixels). The TEM samples were prepared on a 400-mesh Cu grid with thin carbon film cleaned by glow discharge for 45 s. Five microliters of freshly prepared TiO_2_ aqueous solution was deposited on the dull side of the cleaned grid and excess solution was siphoned using a filter paper after 1 min of sample deposition. The sample grid was allowed to dry inside a chemical fume hood and imaged within 24 h of preparation.

Hydrodynamic diameters were measured in ultrapure water and in culture medium at room temperature. The water dispersion of the TiO_2_ sample in LAL reagent water (Lonza, Walkersville, MD, USA) was prepared using a NIST protocol (NIST special publication 1200-3) as a foundation, optimized for the TiO_2_ sample, followed by BSA coating for preparation of dispersion in biological media (NIST special publication 1200-4). TiO_2_ was suspended at 10 mg mL^−1^ by sonicating (Branson Ultrasonics Sonifier Cup Horn Sonicator, Danbury, CT, USA) for 2 h with 80% on and 20% off in a circulating water bath at 4 °C. The hydrodynamic size was assessed using a HydroSV dispersion unit in a Malvern Mastersizer 3000 (Worcestershire, UK) with laser diffraction. Varying sample concentrations (measured as obscuration value) and stirring speeds were investigated to determine the most appropriate conditions for the hydrodynamic analysis. The conditions used to conclude the hydrodynamic size were a 2.6% obscuration value and ~1400 rpm stirring speed at 37 °C. Samples were kept in a temperature-controlled water bath at 37 °C. All other tested conditions skewed the results either towards smaller or larger dimensions. A minimum of three measurements was taken for each sample. The zeta potential of the diluted sample of the TiO_2_ dispersion was measured in a disposable DTS1070 cuvette using a Malvern Zetasizer Nano ZS (Worcestershire, UK) fitted with a 633 nm laser. Measurements were taken with 900 µL of sample containing 100 µg mL^−1^ TiO_2_ in water, 2.5, 5, or 10 mM NaCl solutions.

### 2.2. Cell Lines and Treatment Conditions

The human cell lines Caco-2 and HepG2 were obtained from the American Type Culture Collection (Manassas, VA, USA). The cells were cultured in growth media as recommended by the supplier and routinely maintained at 37 °C in a humidified 5% CO_2_ atmosphere. Cells were seeded in plates (5 × 10^5^ cells per plate) and subsequently treated with 10 or 100 µg mL^−1^ TiO_2_ for 24 or 72 h (Under these conditions, TiO_2_ was not cytotoxic in either of the two cell lines). Untreated cells at each time point were used as controls. At appropriate times, cells were harvested and processed for further analysis.

### 2.3. Evaluation of TiO_2_ Cellular Uptake

TiO_2_ internalization in cells was analyzed by TEM. Caco-2 cells were grown in the recommended cell line medium, harvested by trypsinization, and adjusted to a suspension containing 2.5 × 10^5^ cells mL^−1^. A 200 µL sample of this cell suspension was further seeded per well in an 8-well glass slide (Lab-Tek^®^II Chamber slide system, Thermo Scientific, Waltham, MA, USA). The cells were grown for a period of 24 h, after which the growth medium was removed and replaced with a medium containing TiO_2_, and the cells were incubated for another 24 h. A TiO_2_ concentration of 100 µg mL^−1^ was used for the exposure studies. After 24 h treatment, the medium from the cells was removed and the cells were washed twice with 1× PBS following application of 4% neutral buffered formalin for 15 min at 4 °C. Then, the cells were rewashed with PBS twice and excess PBS was removed. After treatments and washing, samples were prepared following acetonitrile’s serial block face gradient of acetonitrile to dehydrate and infiltrate the samples [52]. After completing the fixation and staining procedures, the samples were dehydrated through an increasing gradient series of acetonitrile washes (20%, 50%, 70%, 90%, and 2× 100%). Then, they were infiltrated for 4 h in 33% Durcapan resin in acetonitrile, 4 h of 66% Durcapan resin in acetonitrile, and overnight in 100% Durcapan resin. The following day, the samples were then placed into molds containing fresh 100% Durcapan and polymerized for 2 days at 60 °C. Once polymerized, the samples were excised from the plastic and mounted on a plexiglass rod with superglue and sectioned at 50–70 nm using a Leica EM UC6 ultramicrotome (Leica Microsystems, Wetzlar, Germany). The sections were then placed in a JEOL JEM-2100 electron microscope for both TEM and elemental analysis by EDS. Regular TEM images were observed at 200 keV at various magnifications depending upon the desired views of the samples. Images of interest were recorded using a TVIPS TemCam-XF416 CCD camera (Tietz Video and Image Processing Systems GmbH, Gilching, Germany).

Elemental analysis was accomplished using the JEM-2100 in STEM mode, where images were recorded by scanning the specimen with an electron probe, rather than by imaging with a single wide beam over the entire sample. The grids were made conductive by carbon coating them on both sides using an Edwards Auto 306 carbon evaporator (Edwards Vacuum, Burgess Hill, UK) to prevent inherent charging from accumulating and burning the sections. The scanning was accomplished using the JEOL brightfield STEM detector (EM-24511SIOD Scanning Image Observation DEV) (JEOL USA, Peabody, MA, USA) mounted on the JEM-2100. Once the STEM mode was aligned correctly and the specimen was continuously being scanned with the microscope set to 20 kX and the electron probe size set to A1, the EDS X-ray detector (Oxford 80T X-Max^N^, Oxford Instruments, Tubney Woods, UK) was inserted into the microscope. Using Oxford Aztec software Aztec 3.1 SP1 (Service Pack 1) to control the STEM detector, the Aztec software was then used to record an X-ray spectrum at each pixel in the specimen image. By continuously scanning the image, the software accumulated and recorded significant signals from the spectra at each pixel. The software then used the different characteristic X-ray energies to identify specifically the elements in the sample at each pixel. This allowed the software to map the identified elements over the recorded EM image with each element of interest shown in a different color. In addition, the overall spectrum of the entire image was calculated as a summary and saved to indicate all the elements observed in the region that were recorded by the software. The results for titanium and oxygen are shown as maps along with the scanned EM image.

### 2.4. Cytotoxicity Assessment

An initial toxicity study was conducted to choose the range of subtoxic doses to be used in our studies. Cell viability was determined by the Promega CellTiter-Glo^TM^ luminescent cell viability assay (Promega; Madison, WI, USA). This assay is a homogeneous method of determining the number of viable cells in culture based on the quantitation of ATP present, which signals the presence of metabolically active cells. Cells were seeded in 96-well plates (n = 10), grown to 85% confluency, and treated with TiO_2_ (5–200 µg mL^−1^) for 24 or 72 h (prepared on treatment day), prepared for analyses according to the manufacturer’s protocol, and then analyzed according to the instructions provided by Promega. Of note, TiO_2_ does not interfere with CellTiter-Glo. This was investigated using a negative control (no cells) throughout the entire process, with everything remaining consistent. Once all steps were completed as prescribed by the manufacturer, luminescence was measured with a BioTek Cytation 5 plate reader (Santa Clara, CA, USA). Results are expressed as mean ± SD activity. The doses selected for the epigenetic experiments were 10 and 100 µg mL^−1^.

### 2.5. Measurement of Global 5-Methylcytosine

Genomic DNA from treated and control cells was extracted using a DNeasy blood and tissue kit (QIAGEN Inc.; Valencia, CA, USA). Analysis of global DNA methylation was conducted using a MethylFlash Methylated DNA Quantification kit (Epigentek Inc.; Farmingdale, NY, USA), an ELISA-based colorimetric assay. The instructions were followed as outlined in the kit using 100 ng of genomic DNA. The methylated fraction of DNA is detected using capture and detection antibodies and then quantified colorimetrically by reading the absorbance in a microplate spectrophotometer BioTek (Cytation 5). Relative quantification was determined by normalizing the readings to the positive control provided with the kit. The results of TiO_2_ exposures on DNA methylation are presented as fold changes relative to the untreated cells.

### 2.6. DNA Methylation PCR Array

The Human Stress & Toxicity PathwayFinder EpiTect Methyl II PCR Signature Array for methylation analysis of a panel of human genes in a 96-well plate format (SABioscience, Qiagen; Frederick, MD, USA) was used to determine the methylation status of selected genes using a real time QuantStudio 7 Flex (Applied Biosystems, Life Technologies, ThermoFisher Scientific, Rockford, IL, USA). As noted by the manufacturer, the panel profiles the methylation status of 22 genes indicative of cellular stress responses and toxicity and is shown to be epigenetically regulated. Quantification of relative DNA copies was carried out by determining the threshold cycle (C_T_). Cycles were programmed as specified in the Methyl-Profiler protocol. The Methyl-Profiler PCR Array Excel-based data analysis template was downloaded from the SABioscience website at: http://www.sabiosciences.com/dna_methylation_analysis.php (accessed 15 January 2023). C_T_ values were entered into the raw data table, and the results were automatically determined for each gene analyzed, as described in detail in the Methyl-Profiler DNA Methylation PCR Array System User Manual (SABioscience, Qiagen). Percentage methylation was calculated using the appropriate C_T_ values. Heatmap analysis was performed using GraphPad Prism (9.5.1).

### 2.7. RNA Extraction and Quantitative Reverse-Transcription PCR (qRT-PCR)

Total RNA was isolated from the treated and control cells using miRNeasy Mini kits (Qiagen). RNA (4 μg) was reverse-transcribed using random primers and high-capacity cDNA reverse-transcription kits (Life Technologies, Grand Island, NY, USA). The expression of *DNMT1*, *DNMT3a*, *DNMT3b*, *MBD2*, and *URHF1* genes was determined by qRT-PCR using the TaqMan gene expression assays (Life Technologies, ThermoFisher Scientific; Rockford, IL, USA): DNMT1 (Hs00154749_m1), DNMT3a (Hs01027166_m1), DNMT3b (Hs00171876_m1), DNMT3L (Hs01081364_m1), MBD2 (Hs00969366_m1), and URHF1 (Hs01086727_m1). Also, *DNAJC15*, *INSIG1*, *BNIP3*, *SCARA3*, *and TP53* genes: DNAJC15 (Hs00387763_m1), INSIG1 (Hs00356479_m1), BNIP3 (Hs00969291_m1), SCARA3 (Hs00939871_m1), TP53 (Hs01034249_m1). The relative amount of each mRNA transcript was determined using the 2^−ΔΔCt^ method [52] with glyceraldehyde-3-phosphate dehydrogenase GAPDH (Hs02758991_g1) as a reference.

### 2.8. Statistical Analysis

Significant differences (*p* < 0.05) between groups were evaluated using an unpaired 2-tailed Student’s *t*-test. All experimental values were normalized to those of the control samples. Graphs represent the means ± SD.

## 3. Results

### 3.1. TiO_2_ Characterization

**Figure 1:**
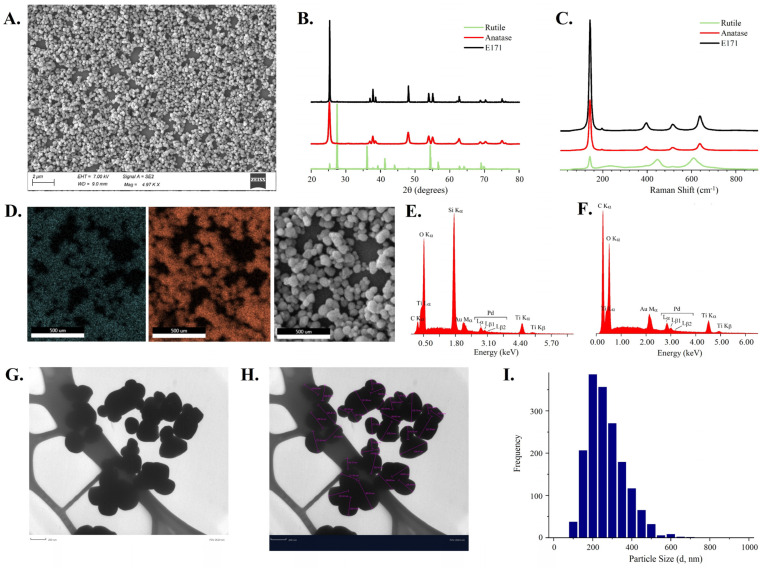
Physicochemical characterization of TiO_2_. (**A**) SEM of TiO_2_; (**B**) XRD pattern for rutile TiO_2_, anatase TiO_2_, and the tested TiO_2_ sample; (**C**) Raman spectra for rutile, anatase, and TiO_2_ sample; (**D**) SEM–EDS mapping of TiO_2_. Blue (left) represents titanium, red (middle) represents oxygen, and the right shows sample region mapped for compositional elements; (**E**,**F**) SEM-EDS spectra of sample in silicon and carbon substrates, respectively; (**G**–**I**) TEM image of TiO_2_ sample; representation of size measurement performed manually on same image (scale bar = 200 nm); size distribution histogram of particle size distribution of TiO_2_ determined using TEM images with n = 1663.

The TiO_2_ sample was analyzed for morphology, crystalline structure, particle size distribution, elemental composition, hydrodynamic diameter, and zeta potential. The SEM image in Figure 1A of the powder TiO_2_ sample shows that it is comprised of generally spherical nanoparticles of heterogeneous size distribution. XRD results comparing the sample with the XRD patterns of anatase and rutile standards demonstrate that the crystalline form of the TiO_2_ sample is anatase (Figure 1B). Additionally, the Raman spectral signals confirm that the sample is in the anatase form (Figure 1C). Figure 1D–F show the elemental mapping of the sample for titanium (Figure 1D) and oxygen (Figure 1E) for the mapped region of the sample shown in Figure 1F. Based on SEM-EDS, the TiO_2_ sample showed the predominant presence of Ti and O. Small amounts of Au, Pd, and C were also seen due to sample preparation methodology. For samples prepared in Si and C substrates, significant intensities corresponding to Si and C, respectively, were also observed. Therefore, elements observed other than Ti and O were either due to sample preparation or substrate material. No other elements were detected. XRD studies showed that the titanium sample was 98.6% anatase. Heterogeneous size distribution was observed with TEM (Figure 1G,H). The size distribution histogram, shown in Figure 1I, was obtained from analysis with 1663 individual particles and showed an average size of 242.3 ± 93.6 nm; nanoparticles and microparticles were present.

**Figure 2:**
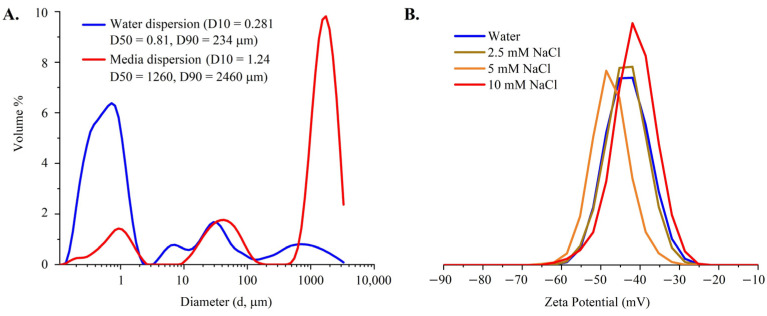
Hydrodynamic size and zeta potential of TiO_2_. (**A**) Average hydrodynamic size distribution of TiO_2_ sample dispersed in water and in media at 37 °C using laser diffraction at obscuration of 2.6% and stirring speed of 1400 rpm. Average sizes based on D-values are also shown. (**B**) Zeta potential of TiO_2_ sample in water (pH 6.4), 2.5, 5, and 10 mM aqueous NaCl solution (pH 6.8).

Hydrodynamic size was determined using laser diffraction. These measurements were taken with an aqueous dispersion of the TiO_2_ sample and with a sample in cell culture medium at room temperature and at 37 °C. Figure 2A shows that the hydrodynamic size distribution of the TiO_2_ sample, measured at an obscuration value of 0.26% and a stirring speed of 1400 rpm, was 0.28 µm (D10), 0.81 µm (D50), and 4234 µm (D90), indicating significant agglomeration in water and severe agglomeration in media. The zeta potential measurement of the sample, as shown in Figure 2B, measured in water (pH 6.4) and 10 mM aqueous sodium chloride (pH 6.8) solutions showed that the sample had an average zeta potential value of −42.4 and −41.6 mV, respectively. Zeta potential in 2.5 mM and 5 mM NaCl solution was −44.1 and −46.8 mV respectively. Based on these zeta potential measurements, it was concluded that the aqueous dispersion of the TiO_2_ sample carried a surface charge, as shown by the zeta potential distribution in Figure 2B.

### 3.2. Evaluation of TiO_2_ Cellular Uptake

**Figure 3:**
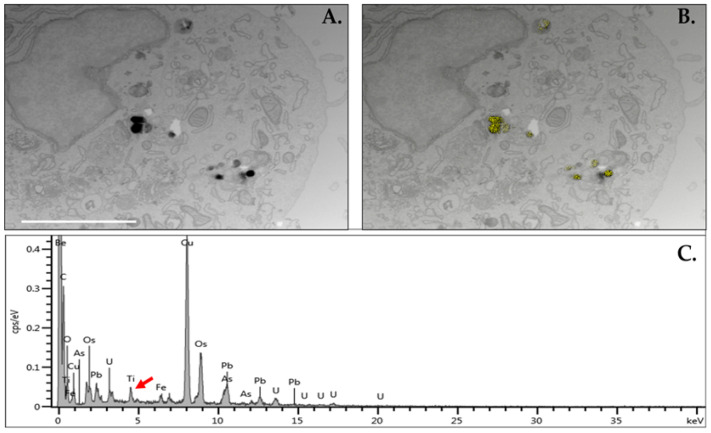
TiO_2_ cellular uptake. (**A**) SEM image of a representative Caco-2 cell exposed to TiO_2_ for 24 h. (**B**) The same image with the locations of the titanium mapped in yellow using energy-dispersive X-ray spectroscopy (EDS) to record the image from the electron microscopy. Both (**A**,**B**) are the same magnification (20,000×). (**C**) The entire EDS spectrum recorded over the entire image. The presence of titanium is indicated by the red arrow. Only the locations of titanium are shown in this figure. White bar equals 2.5 µm.

Using Caco-2 cells as a model cell line, TEM revealed the uptake of the TiO_2_ particles in the cells (dark images) after 24 h (Figure 3A). The presence of the particles was further verified and confirmed by EDS (yellow particles) (Figure 3B). Aztec software recorded the X-ray spectrum and specially identified each element in the image; this further showed the presence of TiO_2_ particles (red arrow) (Figure 3C). The TEM analysis showed that the TiO_2_ particles escaped the endosomes and were present in the cytoplasm, which allowed them to directly interact with cellular organelles.

### 3.3. Cytotoxicity Assessment

**Figure 4:**
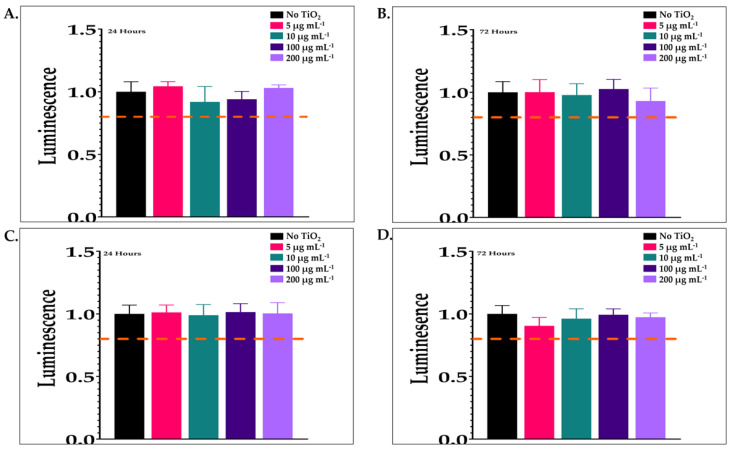
Cytotoxicity assessment. Cell viability was determined using a Promega CellTiter-Glo^TM^ luminescence assay. (**A**) Luminescence of Caco-2 cells exposed to various concentrations of TiO_2_ (0, 5, 10, 100, and 200 µg mL^−1^) for 24 h; (**B**) luminescence of Caco-2 cells exposed to various concentrations of TiO_2_ (0, 5, 10, 100, and 200 µg mL^−1^) for 72 h; (**C**) luminescence of HepG2 cells exposed to various concentrations of TiO_2_ (0, 5, 10, 100, and 200 µg mL^−1^) for 24 h; (**D**) luminescence of HepG2 cells exposed to various concentrations of TiO_2_ (0, 5, 10, 100, and 200 µg mL^−1^) for 72 h. The results are expressed as the mean ± SD of three determinations. Error bars indicate standard deviation.

The cytotoxic effects of TiO_2_ exposure (5–200 µg mL^−1^ for 24 or 72 h) were studied in Caco-2 and HepG2 cells using a Promega CellTiter-Glo^TM^ luminescent cell viability assay (Promega; Madison, WI, USA). Cell viability did not decrease in either cell line by more than 10% on average, if any decrease was present (Figure 4). Luminescence values were not significantly different from controls for any tested groups. These results demonstrated that TiO_2_ did not adversely affect cells at any of the concentration or duration assessed to include those selected for the epigenetic experiments. As expected, luminescence was not seen in the negative control (absence of cells).

### 3.4. Global DNA Methylation Analysis

**Figure 5:**
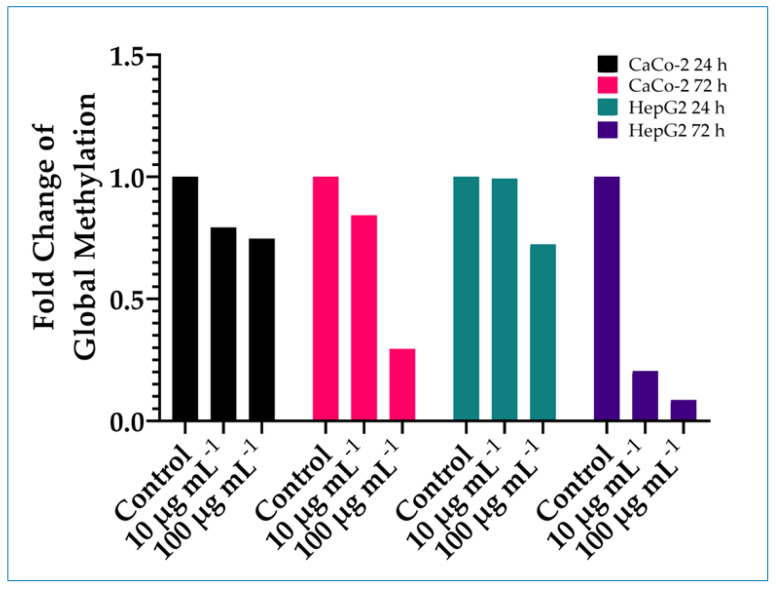
Effect of exposure to TiO_2_ on global DNA methylation. Cells (Caco-2 and HepG2) were treated with 10 or 100 µg mL^−1^ TiO_2_ for 24 or 72 h. Genomic DNA was extracted. Global methylation status was quantified in a microplate-based enzyme-linked immunosorbent assay. Values were derived as fold change in treated cells compared to the untreated cells (controls) in duplicate determinations.

The effects of TiO_2_ exposure (10 or 100 µg mL^−1^ for 24 or 72 h) on global DNA methylation status in the cell lines (Caco-2 and HepG2) was assessed by using a MethylFlash Methylated DNA Quantification kit, an ELISA-based colorimetric assay (Figure 5). Global methylation was decreased (hypomethylation) compared with the control in the treated cells. The effect was concentration- and time-dependent, with the greatest decrease being shown at the extended time point and higher concentration in both cell lines, with several deviations. In Caco-2 cells treated with 10 µg mL^−1^ TiO_2_, the value was higher at 72 h compared to 24 h, and in HepG2 cells treated with 10 µg mL^−1^ TiO_2_, no decrease was observed.

### 3.5. Array Analysis of Gene-Specific DNA Methylation

**Figure 6:**
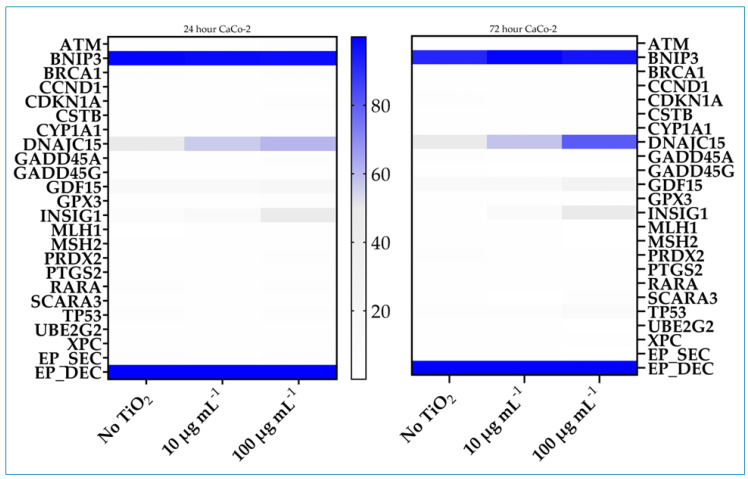
Heatmap of the intensity of promoter methylation of the 22-genes panel in Caco-2 cells. Cells were cultured in the absence or presence of 10 or 100 µg mL^−1^ TiO_2_ for 24 or 72 h. Methylation was assessed by a Methyl-Profiler DNA Methylation PCR System. Color code and intensity indicate level of methylation.

**Figure 7:**
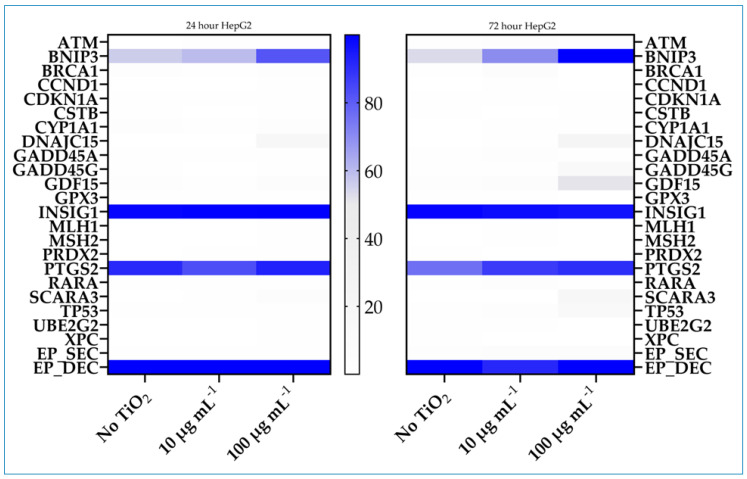
Heatmap of the intensity of promoter methylation of the 22-genes panel in HepG2 cells. Cells were cultured in the absence or presence of 10 or 100 µg mL^−1^ TiO_2_ for 24 or 72 h. Methylation was assessed by a Methyl-Profiler DNA Methylation PCR System. Color code and intensity indicate level of methylation.

The degree of promoter methylation across a defined panel of genes was evaluated in control and cells exposed to TiO_2_ using Methyl Profiler DNA Methylation PCR System technology. The signature panel profiles of genes indicating cellular stress responses and toxicity with a focus on epigenetically regulated genes were confirmed experimentally to show changes in promoter methylation status. Cells were exposed to TiO_2_ for 24 or 72 h at 10 or 100 µg mL^−1^ concentration. Compared to controls, several of the 22 genes in the panel were identified that displayed an increased percentage of promoter methylation, generally increasing with exposure time and concentration. In Caco-2 cells, the genes included *DNAJC15*, *GDF15*, *INSIG1*, and *TP53*; in HepG2 cells, *BNIP3*, *GADD45G*, *DNAJC15*, *GDF15*, *SCARA3*, and *TP53* (Figure 6 and Figure 7). Several genes, *DNAJC15*, *GDF15*, and *TP53*, were affected in both cell lines. Increased methylation in these genes ranged from 16% to 43%, 12% to 50%, and 9% to 16%, respectively, after 100 µg mL^−1^ exposure for 72 h.

**Figure 8:**
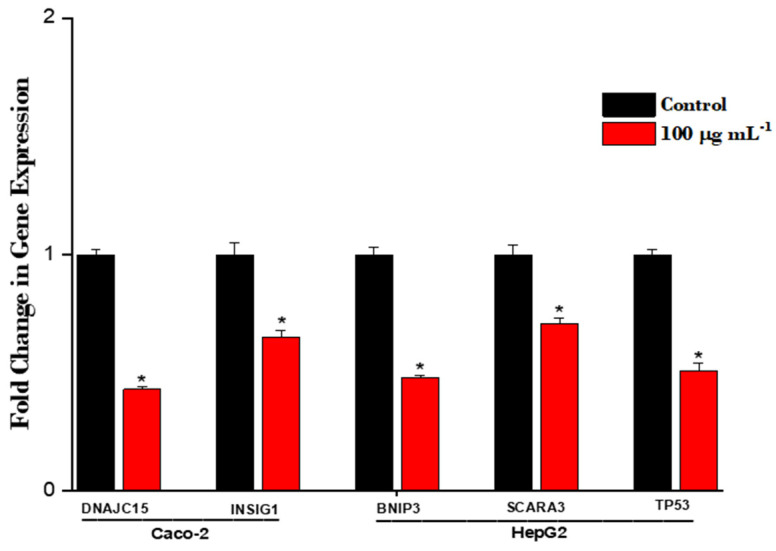
Effect of exposure to TiO_2_ on gene expression. Caco-2 or HepG2 cells were treated with 100 µg mL^−1^ TiO_2_ for 72 h. Expression levels were determined by qRT-PCR using TaqMan gene expression assays. Values were derived as fold change in treated cells compared to the untreated cells (controls) and expressed as means ± SD of three determinations. Error bars indicate standard deviation. * Significant fold change (*p* < 0.05) from control cultures.

Gene expression: To assess whether changes in promoter methylation were associated with gene expression, levels of expression of several of these genes were determined in exposed cells. Decreased expression was observed for each gene (Figure 8).

### 3.6. Effects of TiO_2_ Exposure on Expression Levels of DNMTs, MBD2, and URHF1

**Figure 9:**
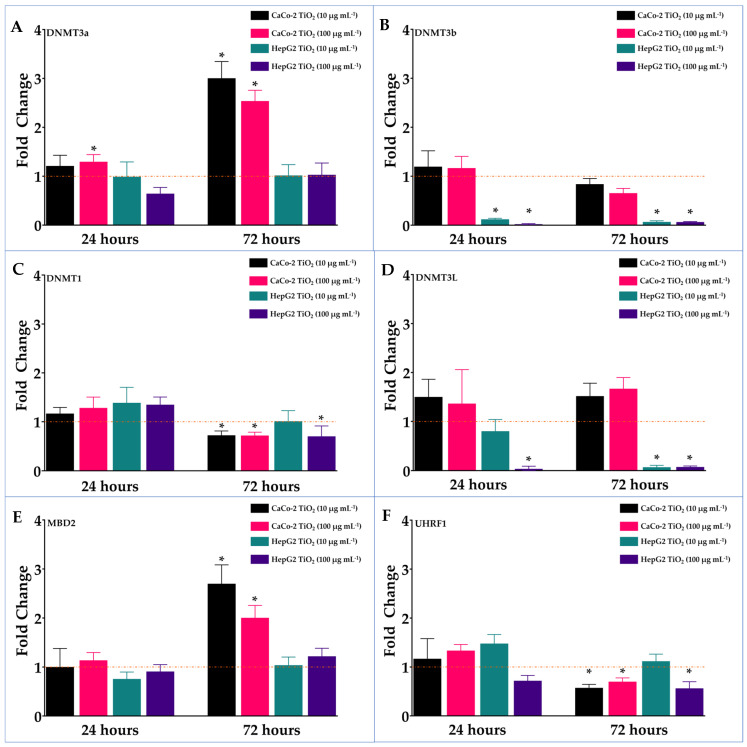
Effect of exposure to TiO_2_ on expression of DNMT3a (**A**), DNMT3b (**B**), DNMT1 (**C**), DNMT3L (**D**), MBD2 (**E**), and UHRF1 (**F**). Cells (Caco-2 and HepG2) were treated with 10 or 100 µg mL^−1^ TiO_2_ for 24 or 72 h. Expression levels were determined by qRT-PCR using TaqMan gene expression assays. Values were derived as fold change in treated cells compared to the untreated cells (controls) and expressed as means ± SD of three determinations. Error bars indicate standard deviation. * Significant fold change (*p* < 0.05) from control cultures.

To investigate further the impact of TiO_2_ exposure, the effects on DNA methylation machinery were assessed. As shown in Figure 9, significant changes in expression levels of the DNMTs occurred after treatment. These alterations varied among the DNMTs and cell lines. Expression of DNMT1 was significantly decreased in Caco-2 and HepG2 cells at 72 h. DNMT3a expression was increased in Caco-2 cells, but not in HepG2 cells. Decreased expression of DNMT3b was found in HepG2 cells. In Caco-2 cells expression levels were not significantly changed. This same effect was observed with DNMT3L expression. Several binding proteins were also affected by TiO_2_ exposure. MBD2 expression was significantly increased in Caco-2 cells at 72 h, but not in HepG2 cells. UHRF-1 expression deceased in Caco-2 cells and HepG2 cells.

## 4. Discussion

DNA methylation is one of the main epigenetic mechanisms. Given the involvement of aberrant DNA methylation in the pathogenesis of an increasing number of disorders and diseases, there is increasing interest in assessing the possible epigenetic toxicity induced by nanomaterials as a critical approach in better understanding the potential risks to human health posed by these materials. Although exposure to TiO_2_ nanoparticle models (including P25) has been shown to alter DNA methylation in several test systems, assessment of a TiO_2_ product to which humans could be exposed in their diet has not been reported. This study investigated the impact of exposure to this material on DNA methylation status in two cell lines relevant to human exposures.

In assessing the effect of exposure to the TiO_2_ material on global methylation in the cell lines, decreased levels were found in the colorectal cells (Caco-2) and liver cells (HepG2). Global hypomethylation is associated with increased chromosome instability and is a distinct hallmark of carcinogenesis [53,54], suggesting potential harm by the TiO_2_ product. Exposure to this material in these cell lines also altered expression levels of *DNMT1*, *DNMT3a*, *DNMT3b*, and *DNMT3L* and binding proteins *MBD2* and *UHRF1*. However, the direction of the gene expression changes depended upon the cell line. Although these results of varying effects were similar to those from studies of TiO_2_ nanoparticles models conducted in several cell types [55,56,57,58,59,60,61], it should be noted that there were differences in several specific alterations observed with this TiO_2_ product compared to those observed in a previous study conducted with the P25 model nanoparticles [61]. While the reasons for these differences are not yet known, they could be due to the substantial difference from the P25 material and the test sample of the current study [16,17,18]. Global hypomethylation was found in most of the cell systems. Although additional data will be needed, the results from this study contribute evidence that the TiO_2_ product can also impact the methylation status in the cell.

Aberrant gene-specific DNA methylation is also critical in dysregulation of cellular function. Studies have reported that exposure to TiO_2_ nanoparticles models alters the methylation status in several genes [55,56,57,58,59,60,61]; however, reports of the effects of a TiO_2_ product to which humans could be exposed in their diet were not found. In the current study, promoter methylation status was examined in a panel of 22 genes that are connected to cellular stress responses and toxicity. In the two cell lines, 7 of the 22 genes identified were affected by exposure to TiO_2_ particles, with three (*DNAJC15*, *GDF15*, and *TP53*) of these seven genes affected in both cell lines. Promoter methylation increased in each of the seven genes. Altered methylation in several genes was also found to be associated with changes in gene expression. In a previous study, promotion methylation of several of the seven genes was also found to be changed in cells by the TiO_2_ nanoparticle P25 reference material, including *DNAJC15*, *GDF15*, *INSIG1*, *SCARA3*, and *TP53* [61]. These genes represent several pathways, suggesting their potential involvement in TiO_2_ toxicity. Gene methylation has been associated with various disease states.

Promoter methylation of *DNAJC15* was observed in both cell lines. The loss of DNAJC15, a mitochondrial protein that belongs to the DNAJ C family of co-chaperones, results in increased mitochondrial respiration and consequently elevated levels of ROS and oxidative stress [62]. Regulation of *DNAJC15* expression involves gene hypermethylation in cancer cells, which has been demonstrated in several cancer cell types, including brain [63], ovarian [64], and melanoma [65]. Promoter methylation of *GDF15* was also observed in both cell lines. GDF-15, a member of the transforming growth factor superfamily [66], regulates cellular responses to stress signals and inflammation and tissue repair after acute injuries. The dysregulation of *GDF-15* expression and signaling pathways is associated with diverse human diseases and cancer progression. *GDF-15* methylation has been suggested to be involved in several cancers, including bladder cancer [67], urothelial cancer [68], and glioblastoma [69]. Promoter methylation of *TP53* also occurred in both cell lines. TP53, a well-known tumor suppressor, regulates the expression of various genes involved in maintaining homeostasis, including those involved in cell cycle regulation, redox homeostasis, DNA replication and repair, and autophagy [70]. Post-translational modifications of *TP53*, including promoter methylation, can result from various forms of cellular stress [71]. *TP53* promoter methylation is associated with various cancers, including liver cancer [72] and leukemia [73].

Promoter methylation of *INSIG-1* was observed in Caco-2 cells. *INSIG-1* (insulin induced gene 1) plays a critical role in the feedback control of lipid synthesis, may be protective against glucolipotoxicity, has combined effects with high glucose and high lipids on beta cell dysfunction [74], and may be involved in linking oncogenic signaling and fuel availability to lipogenesis [75]. Promoter methylation of *INSIG-1* has been shown in gastric cancer [76].

Promoter methylation of *BNIP3*, *GADD45A*, and *SCARA3* occurred in HepG2 cells. *BNIP3* has various cellular functions and has also been shown to be involved in various disease conditions, including myocardial ischemia, autophagy, and apoptosis, and is thereby associated with the pathogenesis of diseases such as cardiovascular disease and cancer [77]. In several cancers, including liver [78], colorectal [79], pancreatic [80], and hematopoietic tumors [81], *BNIP3* methylation has been observed. *GADD45A* is often induced by DNA damage and other environmental and physiological stress signals. It also has a role in development and carcinogenesis [82]. Promoter methylation of *GADD45A* has been observed in several types of human cancers, including breast cancer [83] and lung cancer [84]. Analysis of promoter status of cell cycle-related genes in the acute promyelocytic leukemia cell line NB4 found that several genes, including *GADD45A*, were methylated and could be demethylated by the hypomethylating agent arsenic trioxide [85]. Additionally, in classical Hodgkin lymphoma cell lines, the antitumor effect of the demethylating agent decitabine was associated with induction of genes that negatively regulate cell cycle progression, including *GADD45A* [86]. The *SCARA3* (scavenger receptor class A, member 3) gene, encoding a macrophage scavenger receptor-like protein, is alternatively referred to as a cellular stress response gene, since it protects cells by scavenging reactive oxygen species (ROS) and other harmful oxidation products [87]. Myeloma progression was associated with decreased *SCARA3* mRNA expression in a retrospective analysis of human clinical myeloma samples [88]. *SCARA3* could be considered a tumor-repressor gene in multiple myeloma, since myeloma patients have increased markers of systemic oxidative stress. Additionally, increased *SCARA3* mRNA expression was observed in a myeloma cell line, MM.IS, treated with the DNA methyltransferase inhibitor aza-dC, indicating epigenetic regulation. In hepatocellular carcinoma *SCARA3* was downregulated and shown to act as a tumor suppressor [89]. Promoter methylation of *SCARA3* and its downregulation has also been demonstrated in prostate cancer [90]. A consistent increase in DNA methylation with increased disease duration was observed in a cohort of patients with type 2 diabetes mellitus. The authors suggested that this epigenetic alteration contributed to the pathogenesis and course of the disorder [91].

The observed effects of exposure to the TiO_2_ product on DNA methylation may involve oxidative stress. Although most studies implicating oxidative stress induced by ROS in the toxicity of TiO_2_ have been conducted with TiO_2_ nanoparticle models [43], reports have now shown that food-grade TiO_2_ can also induce oxidative stress [9,14,44,45]. There is increasing evidence demonstrating the influence of oxidative stress on DNA methylation [46,47,48,49,50,51]. Although the underlying mechanisms are not yet well understood, in several pathologies, including diabetes and colorectal and hepatic cancers, it has been suggested that both hypomethylation and specific hypermethylation can be linked to oxidative stress. Silencing of tumor suppressor genes through promoter hypermethylation in response to hydrogen peroxide treatment has been demonstrated in several in vitro systems [47,92]. Aberrant expression of genes involved in DNA methylation (*DNMT1*, *DNMT3a*, and *MBD4*) was detected in a model of chronic oxidative stress-induced malignant transformed human kidney epithelial cells [93], suggesting this pathway as a possible molecular link between oxidative stress and DNA methylation regulation. Additionally, in one report involving TiO_2_ nanoparticle model material the relationship between oxidative stress and DNA methylation involved in the toxic effect of TiO_2_ was examined [55]. Treatment of A549 cells with TiO_2_ nanoparticles resulted in aberrant hypermethylation of PARP-1 (poly(ADP-ribose) polymerase 1). Using a methyltransferase inhibitor and a ROS scavenger, evidence was presented supporting an association between DNA methylation of this gene and oxidative stress. The utility of the DNA methyltransferase inhibitor 5-azacytidine in exploring the relationship between DNA methylation and oxidative stress was demonstrated in another study in which this inhibitor was shown to modulate the expression of the antioxidant enzyme superoxide dismutase, thereby affecting the cellular defense system [94]. The same study showed that the effects of ROS on genomic stability in the cell can also involve impaired telomerase activity. Further studies will however be needed to define the causal pathway(s) connecting exposure to the TiO_2_ product and altered DNA methylation.

## 5. Conclusions

In conclusion, findings from this study clearly demonstrate the impact of exposure to a TiO_2_ product on DNA methylation in human cells, supporting the potential involvement of this epigenetic mechanism in the harmful effect of the TiO_2_ material. Global hypomethylation was observed. Identifying the specific genes with altered promoter methylation status provides additional insight into possible pathways of TiO_2_-induced cellular responses. Changes in the expression of several genes were shown. Further studies will be needed to identify and functionally characterize other genes affected by DNA methylation alterations in response to this TiO_2_ material. A better understanding of the epigenetic effects of TiO_2_ products, as they provide information on their biological properties, should promote risk assessment and safe-use practices of this type of material.

## Data Availability

Data are contained within the article.
